# Inside the biology of early T-cell precursor acute lymphoblastic leukemia: the perfect trick

**DOI:** 10.1186/s40364-021-00347-z

**Published:** 2021-12-20

**Authors:** Francesco Tarantini, Cosimo Cumbo, Luisa Anelli, Antonella Zagaria, Giorgina Specchia, Pellegrino Musto, Francesco Albano

**Affiliations:** 1 Department of Emergency and Organ Transplantation (D.E.T.O.) - Hematology and Stem Cell Transplantation Unit, University of Bari “Aldo Moro”, P.zza G. Cesare, 11, 70124 Bari, Italy; 2 School of Medicine, University of Bari “Aldo Moro”, 70124 Bari, Italy

**Keywords:** ETP-ALL biology, Lymphoid-myeloid potential, Molecular pathogenesis.

## Abstract

Early T-cell precursor acute lymphoblastic leukemia (ETP-ALL) is a rare, distinct subtype of T-ALL characterized by genomic instability, a dismal prognosis and refractoriness to standard chemotherapy. Since its first description in 2009, the expanding knowledge of its intricate biology has led to the definition of a stem cell leukemia with a combined lymphoid-myeloid potential: the perfect trick. Several studies in the last decade aimed to better characterize this new disease, but it was recognized as a distinct entity only in 2016. We review current insights into the biology of ETP-ALL and discuss the pathogenesis, genomic features and their impact on the clinical course in the precision medicine era today.

## Background

Early T-cell precursor acute lymphoblastic leukemia (ETP-ALL) was first described in a cohort of pediatric patients as a distinct subgroup of T-ALL, based on a peculiar gene expression profile (GEP) and immunophenotypic characteristics [1]. ETP-ALL accounts for approximately 16% of childhood and 22% of adult T-ALL, and is generally associated with high genomic instability, poor outcomes and refractoriness to standard chemotherapy [2, 3]. However, recent evidence shows few differences in terms of survival and prognostic significance compared to T-ALL [4]. Since its seminal description, ETP-ALL has gained increasing interest in light of its adverse clinical features and complex biology. It is now considered as a stem cell leukemia at the crossroads of the lymphoid and myeloid fates: the perfect trick [5, 6]. This review aims to discuss current insights into the biology of ETP-ALL, to probe its complex pathogenesis, genomic features and their impact on the clinical course.

### The cell of origin and leukemogenic patterns

The original pathogenic hypothesis of ETP-ALL envisaged the oncogenic transformation of normal ETPs [1]: immature thymocytes capable of migrating from the bone marrow (BM) to the thymus, retaining both the lymphoid and myeloid differentiation potential, thus indicating a direct derivation from hematopoietic stem cells (HSCs) [7, 8]. Coustan-Smith and colleagues identified the ETP as the cell of origin based on GEP, revealing comparable profiles between normal and leukemic cells [1]. Moreover, they demonstrated that the ETP-ALL gene expression signature resembled that of murine ETP [1]. Nevertheless, their hypothesis is still a matter of debate.

ETP-ALL may arise from slightly more differentiated T cells; in the stepwise development of thymocytes, the potential for lymphoid and myeloid differentiation is retained beyond the ETP until the CD4 and CD8 double-negative 2 (DN2) stage [8, 9]. Exceeding this concept, the sleeping beauty transposon system advanced by Berquam-Vrieze and colleagues, an approach in which transposon mutagenesis is initiated at different developmental time points along the T-cell lineage, suggested that ETP-ALL may develop from late CD4+CD8+ T cells [10]. On the other hand, detailed comparison of the global transcriptional profile of ETP-ALL with both normal HSC and leukemic myeloid stem cells revealed similar results, whereas no correlations with normal human ETPs were found [5]. These findings demonstrate that ETP-ALL is a neoplasm of a less mature hematopoietic progenitor or stem cell, that undergoes arrest at a very early maturational stage while retaining the capacity for myeloid differentiation.

To date, even if both the transcriptional and mutational landscapes have been extensively described, there is no univocal hypothesis about the leukemogenic patterns of ETP-ALL. Nevertheless, the high prevalence of mutations in genes involving cytokine receptors and RAS signalling, hematopoietic development and histone modification has been the basis of various studies [5]. Approximately 20% of pediatric ETP-ALL cases harbour activating mutations in the *interleukin-7 receptor* (*IL7r*) or the downstream *Janus kinases JAK1* and *JAK3* genes. It was demonstrated that *Il7r* mutants are capable of blocking thymocyte differentiation at the DN2 stage and inducing ETP-ALL in transplanted mice [11]; moreover, the concomitant introduction of *Runx1* and *Jak3* mutations in hematopoietic stem and progenitor cells in mice gave rise to T-ALL with the ETP phenotype [12].

Furthermore, mutations in *IL7r* activate STAT5, thus suggesting that activation of the JAK/STAT pathway in ETP-ALL can be independent of the presence of *JAK/STAT* mutations, as confirmed by further evidence [13]. This latter observation opens a therapeutic window for JAK inhibition [13] and suggests that ETP-ALL may require continual IL7r signaling for maintenance and leukemic growth [11].

Moreover, altered IL7r signaling may be involved in the maturation block of thymocytes, by regulating the expression of the oncogenic transcription factor *LMO2* [13]. Experimental data showed that *LMO2* and LYL1 co-expression is frequent in ETP-ALL, and is critical for the LMO2-dependent upregulation of a stem cell-like gene signature, aberrant self-renewal of thymocytes and consequent generation of T-cell leukemia [14]. The central role of IL7r was further addressed in a murine *Zeb2* (a transcription factor of the zinc-finger E-box-binding family) gain of function model, demonstrating an upregulated *Il7r* expression in immature T and ETP-ALL cells [15].

Inactivating mutations in components of the epigenetic regulator polycomb repressive complex 2 (PRC2), such as EZH2, SUZ12, EED, are frequently detected in ETP-ALL and are associated with aberrant RAS signalling in response to the loss of the H3K27me3 repressive histone mark. This leads to activation of gene transcription via recruitment of the bromodomain and extra terminal (BET) proteins [5, 16]. In a murine model of hematopoietic progenitors with overexpressed oncogenic mutant *Nras* and deleted *Cdkn2a* (of note this is uncommon in ETP-ALL) abrogation of either *Ezh2* or *Eed* led to a shorter ETP-ALL latency and deregulation of growth and survival signalling [17]. *Ezh2* inactivation was associated with a stem cell gene expression profile, and deregulation of cytokine expression and their receptors [18]. Moreover, abrogation of *Ezh2* accentuated *Ras* deregulation and increased *Stat3* phosphorylation, thus reinforcing the role of the JAK/STAT pathway as a therapeutic target [18]. In line with the above data, STAT3 hyperactivation was highly dependent on IL6 and IL7 stimulation [13, 18].

Concurrent mutation of *EZH2* and the transcription factor *RUNX1* is a relatively common event in ETP-ALL [5]; experimental data in normal ETP showed that inactivation of either of the two genes did not affect cell development, whereas inactivation of both genes resulted in expansion of the ETP pool and blocked differentiation [19]. Interestingly, even if the transcriptional profile of these mutant ETP cells was comparable to that of ETP-ALL, no signs of leukemia development were detected, indicating that further oncogenic hits are needed to complete leukemogenesis.

Coherently, in *Runx1/Ezh2* double mutant mice the acquisition of *FLT3* internal tandem duplications (ITD) activating mutations (a frequent event in ETP-ALL) led to leukemia development [19, 20]. Further observations in *FLT3* mutated ETP-ALL lacking clonal T cell receptor (TCR) rearrangement seem to confirm that leukemic transformation takes place at the ETP level [20]. *FLT3*-ITD, too, provides a further molecular marker for target therapy. In the models mentioned above, different cell populations transforming into ETP-ALL were used: DN2 thymocytes, hematopoietic progenitors and ETP, respectively [11, 18, 19]. These data do not only reflect the high and complex genetic heterogeneity of this leukemic subtype but also reinforce the open debate about the cell type in which the leukemic transformation begins.

### Immunophenotypic signature

**Fig. 1:**
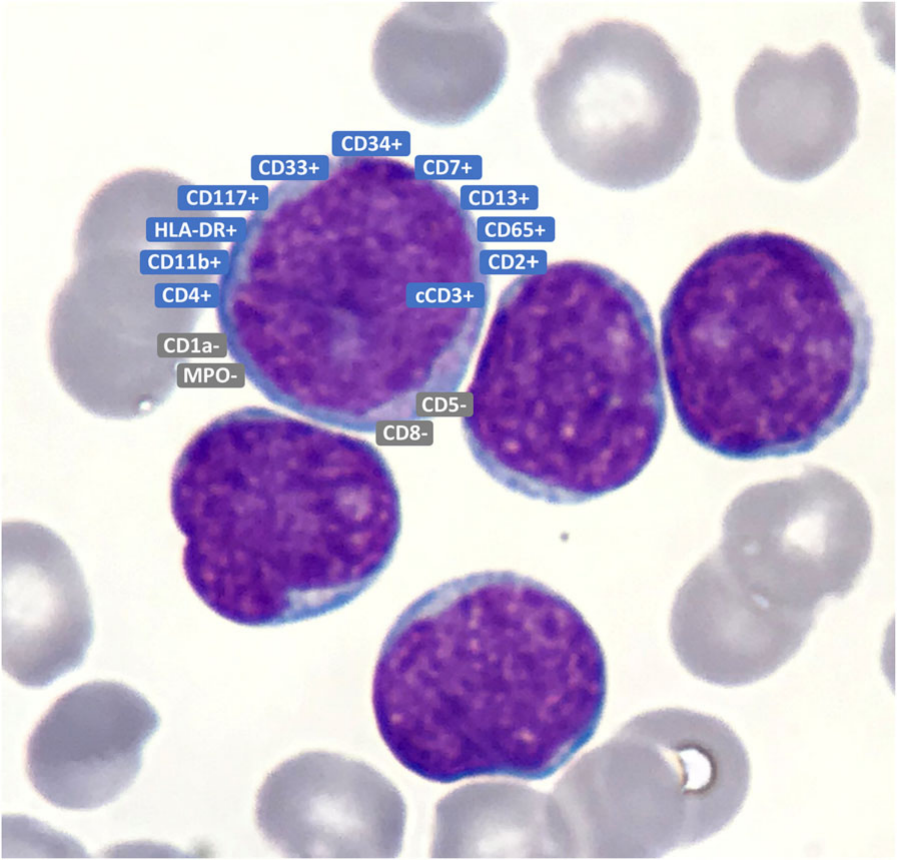
Typical ETP-ALL immunophenotypic profile

Immunophenotypic characterization is a cornerstone in the diagnosis of ETP-ALL. This typically features the expression of CD2, CD7 and cytoplasmic CD3, with at least one stem cell (CD34, CD117) and/or myeloid markers (CD13, CD33, HLA-DR, CD11b, CD65) in at least 25% of leukemic cells, together with negative or weak expression (<75%) of CD5, and the absence of CD1a and CD8 (Fig. 1) [1]. Nevertheless, the correct distinction of ETP-ALL is still a challenge, especially since no specific cytomorphological features have been observed [21].

Scoring schemas based on refined immunophenotypic features have been used to distinguish ETP-ALL from other leukemias in both pediatric and adult settings [22–24]. Of note, very few cases have been reported to co-express B lymphoid markers, indicating that leukemogenesis may possibly initiate at an even more immature precursor stage of ETP, retaining some degree of B lymphoid potential [25, 26].

Moreover, the stringent immunophenotypic signature of ETP-ALL has been addressed by the definition of a “near-ETP-ALL”, expressing CD5 at higher levels and a comparable gene expression profile, suggesting a slightly more mature transforming cell [27]. Interestingly, CD5 expression had been central in correctly identifying all ETP-ALL cases: to recognize ETP-ALL, the authors did not include CD5 but relied on CD7+, with CD34+ and/or CD13+/CD33+, whereas CD1, CD4, CD8 were negative [28]. This immunophenotypic signature allowed the identification of cases with ETP-ALL GEP, with 94% specificity [28]. All these observations point out, on one hand, the importance of immunophenotypic characterization as a practical tool to correctly delineate ETP-ALL cases; on the other, the cited exceptions to the typical signature may reflect the fine line separating one leukemic subtype from another, emphasizing the role of GEP and genomics in the characterization of biologically similar diseases.

### Chromosomal aberrations

No clear association with specific chromosomal abnormalities can be found in ETP-ALL. Available data from various studies mirror the high genomic instability of the disease. Overall, ETP-ALL is reported to have a higher likelihood of harbouring cytogenetic lesions than T-ALL (Table 1); a slightly higher frequency of deletion 13q was reported [1, 3]. A translocation t(2;14)(q22;q32) has been described and associated with the deregulation of *ZEB2* expression, suggesting its possible role in leukemogenesis [15]. Recently, a study on acute leukemias of ambiguous lineage showed the recurrence of 14q32 rearrangements resulting in the activation of *BCL11B*, co-occurring with *FLT3*, *DNMT3A*, *TET2* and *WT1* variants. GEP identified a specific expression signature related to *BCL11B* activation with significant downregulation of its targets, providing a novel biomarker for a new entity among immature acute leukemias [29].

**Table 1:** Chromosomal aberrations and mutational landscape occurrence in ETP-ALL and non ETP-ALL. Marks indicate the disease subtype in which the alteration is more frequent

		ETP-ALL	Non ETP-ALL
**Chromosomal aberrations**	13q deletion	⎫	
	14q32 rearrangements	⎫	
	*LMO1/2*, *TLX1*, *TLX3*, *STIL* rearrangements		⎫
	*KMT2A* rearrangements	⎫	
	*TCR* rearrangements	⎫	
	*CDKN2A/B* deletion		⎫
**Mutational landscape**	*RUNX1*, *IKZF1*, *ETV6*, *GATA3*, *EP300* (hematopoietic development)	⎫	
	*BRAF*, *FLT3*, *IGFR1*, *JAK1*, *JAK3*, *KRAS*, *NRAS*, *IL7r* (RAS and cytokine receptor/JAK-STAT signalling)	⎫	
	*EED*, *SUZ12*, *EZH2* (histone-modifying genes)	⎫	
	*DNM2*, *ECT2*, *RELN* (lymphoid development)	⎫	
	*NOTCH1* and *FBXW7*		⎫
	*FLT3*	⎫	
	*DNMT3A* and *FAT3*	⎫	
	*NPM1*	⎫	

Apart from this, ETP-ALL exhibits a lower frequency of classic recurrent rearrangements (i.e., *LMO1/2, TLX1, TLX3, STIL*) associated with T-ALL, confirming its distinct nature [4]. Other studies have reported a consistent percentage of *KMT2A* rearrangements, while the frequency of TCR locus gene rearrangements seems comparable to that of T-ALL [4, 30]. A whole-genome sequencing analysis further confirmed and refined these findings, demonstrating multiple complex rearrangements in isolated cases and a high prevalence of breakpoints in coding genes with a key role in hematopoiesis and leukemogenesis (i.e., *MLH3* and *RUNX1*) [5]. This latter may result in loss of function of the involved genes or occur as a part of complex translocations resulting in the formation of novel chimeric fusion proteins [5]. In the same cohort, the occurrence of deletions of chromosome 9 encompassing the CDKN2A/B tumour suppressor genes was less frequent as compared to non-ETP-ALL [5]. Furthermore, frequent copy number variation (CNV) events (both gain and loss of genomic material) can contribute to determining the high genomic instability widely described in ETP-ALL [1].

### Mutational landscape

A comprehensive genomic analysis of pediatric ETP-ALL cases described a highly heterogeneous mutational landscape and failed to identify a unifying genetic alteration [5]. Nevertheless, differences between ETP-ALL and T-ALL are remarkable (Table 1) [31]. ETP-ALL was shown to have enriched mutations in three pathways: loss of function alterations in genes regulating hematopoietic development (i.e., *RUNX1, IKZF1, ETV6, GATA3* and *EP300*); activating mutations driving RAS and cytokine receptor/JAK-STAT signalling (i.e., *BRAF, FLT3, IGFR1, JAK1, JAK3, KRAS*, *NRAS* and *IL7r*); inactivating mutations in histone-modifying genes (i.e., *EED, SUZ12, EZH2*). Furthermore, other genes with an unknown role in tumorigenesis and lymphoid development, such as *DNM2, ECT2* and *RELN*, were frequently mutated [5].

**Fig. 2:**
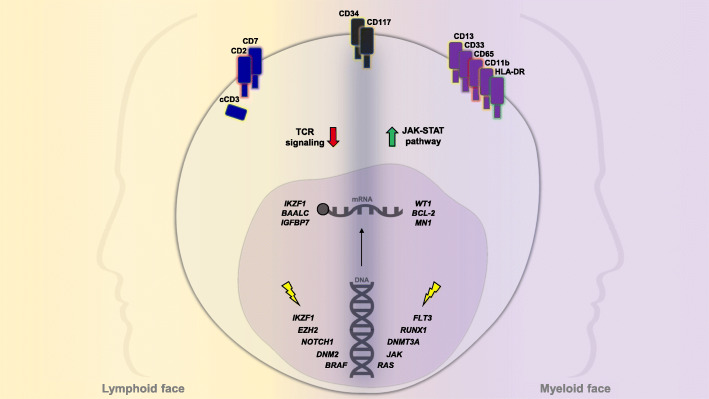
Main biological features showing the double face of ETP-ALL. The lympho-myeloid potential of ETP-ALL cell is summarized, illustrating immunophenotypic, mutational and GEP aspects. Molecular mechanisms not clearly referable to a lymphoid or myeloid context are not reported.

Notably, genes with an established role in both myeloid and B lymphoid malignancies, such as *RUNX1, ETV6* and *IKZF1*, had never before been associated with T-ALL forms. Moreover, mutations in the pathways mentioned above are frequently found in AML [32], emphasizing the “two-faced” nature of ETP-ALL (Fig. 2).

Subsequent studies in adult ETP-ALL cases further refined the scenario [33]. In detail, compared with non-ETP-ALL cases, ETP-ALL showed a lower frequency of mutations in genes commonly involved in the pathogenesis of ALL, namely *NOTCH1* and *FBXW7* [33]. Conversely, a high rate (35%) of *FLT3* mutations was reported, tyrosine kinase domain (TKD) alterations being slightly more prevalent. This is in line with experimental data in mice showing the development of lymphoid diseases upon transplantation of *Flt3*-TKD BM cells [34]. Furthermore, cases harbouring a *FLT3* mutation exhibited distinct clinical features compared with wild-type (wt) *FLT3* ETP-ALL and, interestingly, a slightly different immunophenotype with a constant expression of CD117, CD34, CD13 and CD2 [33]. Extended characterization of *FLT3* mutated ETP-ALL marked a distinct GEP with a higher expression of *WT1* and downregulated expression of *GATA3* and *IGFBP7* genes as compared to FLT3-wt ETP-ALL [20].

Moreover, TCR rearrangements and *NOTCH1* mutations were frequently lacking in these patients; together with the low *GATA3* expression, these data suggest that the leukemic transformation in *FLT3* mutated ETP-ALL might occur at a stem cell pluripotent stage, reflecting a possibly even more immature nature of this ETP-ALL subgroup [20]. Interestingly, in the first genomic characterization of ETP-ALL in pediatric patients, mutations in *FLT3* and the PRC2 complex components were mutually exclusive [5]. This information may further support a different pathogenesis for *FLT3* mutated ETP-ALL. Moreover, the high frequency of FLT3 mutations in this pathological context is of great clinical interest, given the reported therapeutic efficacy of tested tyrosine kinase inhibitors (TKIs) [20].

A subsequent whole exome sequencing analysis provided further insights into the mutational landscape of adult ETP-ALL [6]. Aside from the detection of mutations in genes involved in both T-ALL (i.e., *ETV6, NOTCH1, DNM2*) and myeloid malignancies (i.e., *NRAS, JAK1, FLT3*), recurrent mutations in two genes, *DNMT3A* and *FAT3*, were reported. Notably, none of these had previously been associated with ETP-ALL. Novel mutations in *FAT1* and *MLL2* were also detected [6]. Strikingly, over 60% of the ETP-ALL cases exhibited a mutation in one of these three genes: *FLT3, DNMT3A, NOTCH1*, again reflecting the lymphoid-myeloid potential of the disease (Fig. 2) [6]. To further emphasize this concept, we underline that *DNMT3A* mutations in adult ETP-ALL, present with a similar frequency to that in AML, were located at the same hotspot and were seen in conjunction with mutations in other epigenetic regulators, thus strongly resembling AML cases [6]. Moreover, an age-dependent occurrence of specific mutations was observed: while *DNMT3A* mutations occurred mainly in older patients, inactivation of the PRC2 complex components was significantly less frequent than in pediatric patients [5, 6]. These observations may further suggest that, like in other hematological malignancies, in adult patients ETP-ALL could arise in the context of age-related clonal hematopoiesis [35, 36]. Nevertheless, there is evidence that a considerable proportion of adult early immature T-ALLs, not fulfilling the immunophenotypic criteria for ETP-ALL, shows mutations in myeloid related oncogenes and tumour suppressor genes (such as *IDH1, IDH2, DNMT3A, FLT3, ETV6* and *NRAS)*, while exhibiting a lower prevalence of typical T-ALL genetic alterations (such as mutations in the *NOTCH1* and *FBXW7* genes). This circumstance stresses the overlapping features of ETP-ALL and immature T-ALL [37].

Lastly, the fascinating genomic scenario of ETP-ALL and the need to identify other potential therapeutic targets prompted the creation of a computational model aiming to identify characteristics associated with the mutational profile of ETP-ALL compared to T-ALL cases [38]. Intriguingly, a high frequency of deletion of nucleophosmin-1 (*NPM1*), commonly mutated in AML, was reported, expanding the spectrum of genetic abnormalities shared between myeloid malignancies and ETP-ALL [39, 40].

### GEP

As mentioned above, the first GEP of ETP-ALL in pediatric patients resembled that of murine ETP [1]. Moreover, compared to T-ALL, the identified ETP-ALL cases exhibited a slightly higher expression of oncogenic transcription factors associated with an immature thymocyte phenotype, namely *LMO1, LYL1* and *ERG* [1]. These findings were subsequently refined, revealing a global transcriptional profile similar to that of HSCs and early myeloid progenitors [5]. Analysis of the GEP pathway demonstrated an enrichment of genes mediating the JAK-STAT pathway but no enrichment of T-cell receptor signalling genes (Fig. 2) [5]. Furthermore, reconstruction of the transcriptional network of ETP-ALL identified 30 gene networks (‘regulons’) mastered by *RUNX1* and *IKZF1* [5]; this may indirectly reflect the lymphoid-myeloid potential of the transforming cell. These data are in line with previous observations associating the mutational landscape of ETP-ALL with that of AML.

GEP performed in adult ETP-ALL added further evidence of the “crossover” potential of ETP-ALL. Genes (*BAALC, IGFBP7*) associated with a stem cell signature and adverse outcome in T-ALL, and typically expressed in myeloid leukemia (*WT1, MN1*), are highly overexpressed in ETP-ALL (Fig. 2) [33]. Moreover, low expression of the transcription factor *GATA3* in a cohort of adult ETP-ALL cases was correlated with enrichment of myeloid/lymphoid progenitor and granulocyte/monocyte progenitor genes, while T cell-specific signatures were downregulated, suggesting a *GATA3* role in the selection of a more immature leukemia-initiating cell [41]. In line with this concept, inactivating mutations of *GATA3* are reported as frequent events in pediatric ETP-ALL [5].

Interestingly, experimental evidence shows that the expression of *BCL-2* is higher in ETP-ALL than in more mature T-ALL, thus showing resemblances to AML, and offering a druggable therapeutic target [42, 43]. In this well-defined transcriptional profile context, it is noteworthy that cross-examination of gene sets associated with ETP-ALL [1] and immature T-ALL [28, 44–46] evinces a high degree of relation, indicating a biological link that exceeds the stringent immunophenotypic criteria.

## Discussion

First described in 2009, ETP-ALL was included as a provisional entity only in the 2016 revision of the “WHO classification of tumours of hematopoietic and lymphoid tissues” [47], recognizing its distinct nature. Intriguingly, over the years, its characterization has revealed a fascinating scenario of biological heterogeneity and ambiguity, which places ETP-ALL at the intersection of the lymphoid and myeloid fate, going beyond the well-defined immunophenotypic criteria required for diagnosis [1].

The recognition of genomic features has allowed the definition of both mutational and transcriptional landscapes, suggesting that ETP-ALL may arise from a very early immature thymocyte progenitor with a stem cell-like transcriptional profile, that retains both a lymphoid and myeloid potential [5]. However, the hypothesis regarding the cell of origin and its leukemogenic pathways is still debated. It should be noted that comparison of GEP analyses of ETP-ALL and immature T-ALL has, on one hand, revealed overlapping features, suggesting the definition of a “near-ETP-ALL” entity presenting with only slight immunophenotypic differences, mainly referred to the expression of CD5. On the other hand, it further confirmed that the origin of ETP-ALL is a T cell progenitor [1]. Nevertheless, it is still unclear at which stage of early cellular differentiation the leukemic transformation occurs. As previously reported, in thymocyte differentiation, the myeloid potential is preserved until the DN2 stage [8, 9]. Notably, the observation of cases of ETP-ALL expressing B lymphoid markers raised the idea that this leukemia may arise from an earlier than ETP progenitor, the so-called “thymic seeding progenitor”, “common lymphoid progenitor”, or “lymphoid-primed multipotent progenitor” [25]. Various studies have addressed this issue, with conflicting results [26, 48, 49]. Recent evidence investigating the 3D chromosomal structures of T-ALL demonstrates that global 3D genome architecture and its alterations may reveal differences between normal T cells, T-ALL and ETP-ALL; in this context, T-ALL and ETP-ALL are described as different “frozen stages” of T-cell development with different gene expression profiles associated with 3D genome alterations [55, 56].

Furthermore, there is evidence that even ETPs might present B lymphoid markers [50, 51]. To date, the debate is still open, and we cannot draw clear conclusions. In line with this uncertainty, consistent differences have been described in the mutational landscape of pediatric and adult ETP-ALL. One such observation regards the mutual exclusivity of *FLT3* mutations and PRC2 inactivation, the former being more common in adult patients and the latter in pediatric cases [5, 20, 33]. This finding may offer a further biological point of interest: is adult ETP-ALL closer to myeloid malignancies than T-ALL? Conversely, is pediatric ETP-ALL closer to T-ALL than myeloid malignancies? The age-dependent occurrence of *DNMT3A* mutations, and the computational model revealing a particular frequency of *NPM1* mutation in adult ETP-ALL cases, seem to emphasize this open question [40]. Considering all these premises, the clinical aggressiveness and poor outcomes historically attributed to ETP-ALL should come as no surprise, as they may reflect an inability to target its highly heterogeneous and ever-expanding spectrum of genomic features [1, 3, 52]. The first therapeutic approaches, based on standard chemotherapy regimens in ALL, showed dismal responses and short relapse-free survival [1]. The introduction of pegylated asparaginase in the induction schemes and allogeneic hematopoietic stem cell transplantation improved the prognosis [3, 22]. Nevertheless, various studies demonstrated no differences in event-free survival and overall survival between ETP-ALL and T-ALL [22, 53]. Notably, based on the aforementioned genomic findings, several targeted therapies are being developed for ETP-ALL (Table 2). The JAK inhibitor ruxolitinib, the anti-BCL2 venetoclax, multikinase inhibitors targeting *FLT3* mutations, BET inhibitors, novel immunotherapies (such as CAR-T, CAR-NK and both monoclonal antibodies and immunoconjugates targeting CD38, CD33 and CD123), are being tested alone or in combination with standard chemotherapy in both newly diagnosed and relapsed/refractory ETP-ALL [13, 19, 20, 52, 54].

**Table 2:** ETP-ALL clinical trials. NA: no data available

	Status	Title	ID CODE	Enrolled Patients
1	Recruiting	Advancing Chemical and Genomic Strategies for Relapsed/Refractory T-ALL and ETP-ALL	NCT04582487	32
2	Recruiting	Study of Decitabine Combined with HAAG Regimen in Newly Diagnosed ETP-ALL/LBL, T/M-MPAL and ALL/LBL With Myeloid or Stem Cell Markers Patients	NCT04446130	100
3	NA	Precision Diagnosis Directing HDACi Chidamide Target Therapy for Adult ETP-ALL	NCT03553238	70
4	NA	Ruxolitinib Plus LVP in Patients With R/R ETP-ALL	NCT03613428	12

## Conclusions

The progressive adoption of ever more specific therapeutic approaches is improving the outcome of ETP-ALL patients, underlining the importance of delving more deeply into the biology of the disease. All these observations explain the need for further studies aiming to better characterize the clinical-biological features of ETP-ALL in both pediatric and adult patients. It remains to be seen if, in the precision medicine era, the potential of new generation technologies will allow the secrets of the perfect trick to be unveiled.

## Data Availability

Not applicable.

## Abbreviations

ETP-ALL: early T-cell precursor acute lymphoblastic leukemia
GEP: gene expression profile
BM: bone marrow
HSCs: hematopoietic stem cells
DN2: double-negative 2
PRC2: polycomb repressive complex 2
BET: bromodomain and extra terminal
ITD: internal tandem duplication
TCR: T cell receptor
TKD: tyrosine kinase domain
TKIs: tyrosine kinase inhibitors
